# Multifocal necrotising scleritis post-MIVS in oligoarticular JIA : exploring plausible deniability

**DOI:** 10.1186/s12348-025-00562-x

**Published:** 2025-12-06

**Authors:** Gaurav M. Kohli, Luxmi Singh, Anam Masood, Pratik Shenoy, Digvijay Chaudhary

**Affiliations:** 1 Department of Ophthalmology, Era’s Lucknow Medical College and Hospital, Lucknow, Uttar Pradesh India; 2 Isha Netralaya, Thane, Maharashtra India; 3 Department of Paediatrics, Era’s Lucknow Medical College and Hospital, Lucknow, Uttar Pradesh India

**Keywords:** Necrotising-scleritis, JIA, MIVS, Polyglactin-suture, Oligoarticular

## Abstract

**Aim:**

To report and discuss the clinical features and management strategies for paediatric surgically induced necrotizing scleritis (SINS) following microincision vitrectomy surgery (MIVS), in a patient with underlying seronegative oligoarticular juvenile idiopathic arthritis (JIA).

**Methods:**

Case report and review of literature.

**Results:**

A 10-year-old boy presented with multifocal areas of painless scleral necrosis following three-port 25-gauge pars plana vitrectomy. The area of scleral necrosis remained localized to the temporal sclerotomy sites, which were closed with 6 − 0 polyglactin sutures. There were no associated clinical signs of suppuration or discharge suggestive of an infectious etiology; the smear from the base of the scleral necrosis showed no microbial growth. Scleral melt remained progressive despite immunosuppressive therapy with intravenous methylprednisolone and cyclophosphamide. Systemically, the patient reported a recent onset of multiple joint pains without any visible joint swelling. The blood investigation returned negative for rheumatoid factor, ANA, and ANCA antibodies. Non-specific inflammatory markers (CRP) were significantly raised. The scleral necrosis stabilized following removal of the polyglactin suture. Scleral patch graft with conjunctivo-tenon flap helped to preserve the globe integrity. No recurrence or further necrosis was observed over twelve months of follow-up.

**Conclusion:**

This is the first report on the clinical presentation of paediatric SINS associated with JIA following MIVS. Prior surgeries, underlying autoimmunity and use of polyglactin suture could have precipitated SINS which was recalcitrant to conventional immunosuppressants. A combined medical and surgical approach was helpful to achieve disease remission.

## Introduction

Postoperative ocular surface inflammation may present either as an exaggerated hypersensitivity reaction to suture material or an infectious/ immune-mediated necrotizing scleritis. While polyglactin (Vicryl) sutures are known to cause hypersensitivity reactions, they have also been implicated in rare cases of surgery-induced necrotising scleritis (SINS), particularly following strabismus and pterygium surgeries [1, 2].

SINS in the pediatric population are extremely rare occurrences, wherein its clinical presentation and response to conventional therapy might differ, considering the constitutional differences compared to the adult population [3]. 

Reports defining clinical characteristics and challenges while exploring therapeutic strategies for Pediatric SINS are scarce. Herein, we describe a pediatric case of multifocal noninfectious scleral necrosis and uveal prolapse following a pars plana vitrectomy procedure. Based on the associated symptoms and serological workup, we found that the child also had an underlying juvenile idiopathic arthritis (JIA), which was oligoarticular with negative serology for rheumatoid arthritis (RA) and antinuclear antibody (ANA). In this report, we discuss the clinical presentation, possible precipitants, and management strategies for pediatric SINS.

## Case history

A 10-year-old male presented with pain and redness in the right eye (RE) following an uneventful cataract surgery, done elsewhere a month ago. The etiology of the cataract remained undetermined. At presentation, there were no systemic symptoms suggestive of an underlying autoimmune or infectious process, specifically, no history of joint pain, stiffness, fever, cutaneous lesions, or urinary complaints. Postoperative visual recovery was poor despite adherence to standard postoperative care.

On examination, the RE demonstrated significant inflammation, including circumciliary congestion, iris bombe, irido-capsular adhesions, and a dense, organized membrane over the intraocular lens (IOL), along with posterior capsular opacification (image 1a). Intraocular pressure (IOP) in the RE was markedly elevated at 46 mmHg by applanation tonometry. Best-corrected visual acuity (BCVA) was light perception in the RE and 6/6 in the left eye (LE). The B-scan ultrasonography of the RE showed a quiet mid and posterior vitreous cavity with a retinochoroidoscleral (RCS) thickness of < 1.2 mm. The LE anterior segment and fundus were unremarkable.

A diagnosis of severe postoperative inflammation with secondary angle-closure glaucoma was established. The patient was initiated on systemic steroids (1.5 mg/kg body weight) and topical corticosteroids, cycloplegics, mydriatics, and anti-glaucoma medications.

Due to poor cooperation and inability to perform laser peripheral iridotomy or capsulotomy, a pragmatic surgical approach was adopted. A 25-gauge three-port pars plana anterior vitrectomy, posterior capsulotomy, and surgical peripheral iridectomy were performed after 1 month of steroid cover. The two temporal sclerotomy ports required 6 − 0 polyglactin (Vicryl) sutures, while the nasal port was left sutureless due to good wound apposition after trocar removal.

Two weeks post-surgery, the pupillary block had resolved, with normalization of IOP and visual improvement to 6/36 in the RE. However, despite the systemic steroids, the patient subsequently developed painless, localized scleral necrosis (“scleral melt”) at both temporal sclerotomy sites. Each lesion measured approximately 2 mm in circumferential extent (image 1b).

**Fig. 1:**
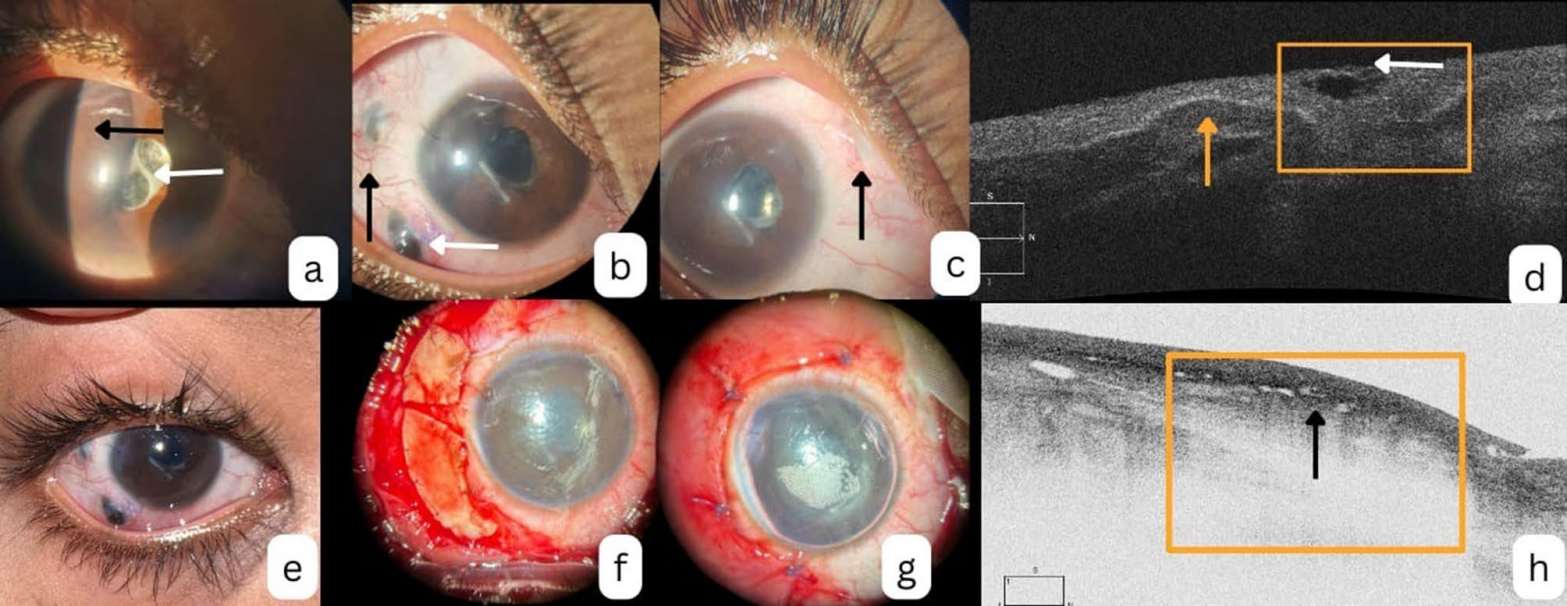
(**a**) Following cataract surgery pupillary block is observed with iris bombe (black arrow) membrane over the intraocular lens 9 white arrow) and opacity behind it (**b**) post operatively 2 weeks- scleral necrosis (white arrow) at the sclerostomy site with adjacent episcleral congestion with dilated conjunctival vessels (black arrow) (**c**) nasal sclerostomy showed healing with no inflammation or thinning (**d**) AS-OCT shows presence of scleral thining (yellow box) with overlying cystic spaces (white arrow), the neighbouring sclera shows normal scleral tissue with overlying conjunctiva (yellow arrow). (**e**) Progressive scleral necrosis despite pulse immunosuppression (**f**) Scleral patch graft applied over the area of scleral thinning. (**g**) Conjunctiva-tenon flap used to cover the scleral patch graft. (**h**) AS OCT following scleral patch graft shows a well-placed scleral graft (yellow box) with overlying healthy conjunctival lamina

Notably, there was no overlying suppuration, and the scleral necrosis was bordered by localized conjunctival inflammation. Dilated conjunctival vessels with arborizing loops were visible adjacent to the melt (image 1b); however, the necrotic zones remained avascular (Fig. 1b). In contrast, the nasal sclerotomy, which was left unsutured, remained quiet with no signs of necrosis or inflammation (image 1c).

Anterior segment optical coherence tomography (AS-OCT) confirmed full-thickness scleral discontinuity at the affected sites, with adjacent tissue disorganization and intrascleral hypo-reflective spaces suggestive of active scleral inflammation (image 1d).

Concurrently, the patient also developed new-onset asymmetrical arthralgia involving the knees, shoulders, and ankles. While joint swelling or deformities were absent, the findings raised clinical suspicion of an evolving systemic inflammatory disorder. The possibility of alternative forms of arthritis, such as septic arthritis and Blau syndrome, was ruled out by the orthopedic team.

Microbial cultures obtained from the base of the scleral necrosis showed no growth on either solid or liquid culture media. Other common infectious diseases like tuberculosis and syphilis were ruled out. A comprehensive serological workup for systemic vasculitis and autoimmune diseases—including antinuclear antibody (ANA), double-stranded DNA, rheumatoid factor, anti-CCP, c-ANCA, p-ANCA, and HLA-B51/B52—yielded no conclusive findings. However, C-reactive protein (CRP) was significantly elevated (> 90 mg/L), indicating active systemic inflammation.

Given the history of ocular surgery and the development of subsequent scleral necrosis corresponding to the site of sclerostomy where polyglactin sutures were used, a diagnosis of surgically induced necrotizing scleritis (SINS) was considered. The presence of polyarthralgia, negative serologies, and elevated inflammatory markers raised the suspicion of an underlying seronegative, oligoarticular juvenile idiopathic arthritis (JIA).

To halt the progression of scleral necrosis, combined pulse immunosuppressive therapy was initiated with intravenous methylprednisolone (30 mg/kg/day for five days) and biweekly cyclophosphamide infusions (1 g/m² in 250 mL normal saline). The patient was subsequently transitioned to oral corticosteroids, tapered over 8–12 weeks, alongside weekly methotrexate (0.5 mg/kg). Progression was noted despite aggressive immunosuppression.

Surgical intervention was performed to retain globe integrity. Polyglactin sutures were removed, conjunctival margins were freshened, and a scleral patch graft was placed, covered by a conjunctivo-tenon advancement flap. A glycerin-preserved donor scleral graft was secured over the necrotic bed, extending 2 mm beyond the defect margin and anchored with 10 − 0 nylon sutures (image 1f). The conjunctiva was repositioned and sutured over the graft (image 1g). Cultures from excised conjunctival margins showed no microbial growth at 72 h. At one month, the graft showed good uptake, with no new areas of thinning clinically. The AS-OCT confirmed a well-integrated hyperreflective graft with posterior shadowing and no residual interlamellar fluid. (image 1h).

**Fig. 2:**
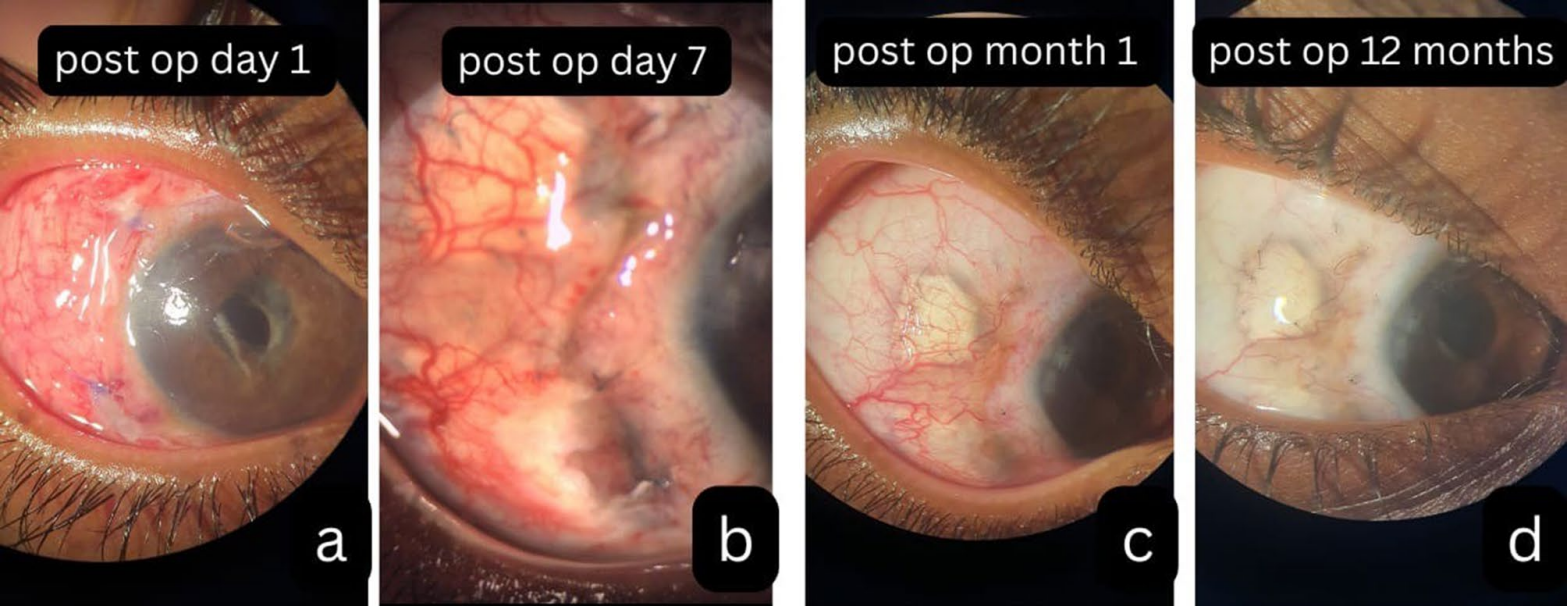
**a**) conjunctival congestion and chemosis in the immediate post operative period obscures underlying graft (**b**, **c**) over one month the congestion has decreased with prominent overlying conjunctival vascularity. **d**) over the next 6 -12 months after completing 3 doses of pulse cyclophosphamide, the conjunctival vascularity over the graft had considerably decreased with stabilization of the scleral melt

Postoperatively, conjunctival chemosis and congestion partially obscured the graft. After completing three cyclophosphamide pulses, vascularity had significantly regressed. With a retained, secure, and stable graft in situ, no reactivation of the disease was seen over 12 months (Fig. 2a-d).

## Discussion

Our report highlights the atypical clinical presentation of post-vitrectomy SINS in a child with coexisting seronegative oligo-articular JIA. Reports of SINS following vitrectomy are exceedingly rare, with only three published cases to date, all in adults [4–6]. Constitutionally, the patients developing SINS may have some form of underlying immune abnormality. Sainz et al. in their series of 10 eyes showed coexisting immune abnormality in 90% of the cases [7]. Similarly, in another series by O’Donoghue et al., an underlying immune anomaly was found in 55% of their patients [8]. Rheumatoid arthritis, seronegative polyarthropathy, Wegener’s granulomatosis, ankylosing spondylitis and ulcerative colitis are some of the well-known associated disorders reported until now [5, 7]. 

Prior literature suggests that SINS can occur after vitrectomy, typically within six weeks, and is often painful [4–6]. SINS has been reported to present with porcelain-white scleral necrosis, pain, and diffuse hyperemia; these findings, though characteristic, were not seen in our case. In contrast, our case featured painless, well-demarcated necrosis with surrounding vascular loops and minimal conjunctival inflammation, but no overlying slough or discharge. The reason for such atypical presentation can possibly be attributed to underlying JIA.

We believe that the clinical presentation of SINS may be altered in the presence of an underlying immune dysfunction. Since not all patients with SINS have underlying autoimmunity, it would be only reasonable to consider that the clinical phenotypes and course of the disease might differ in those with and without autoimmunity. In a multicentric study by Lozano et el, the authors observed that SINS in patients with underlying autoimmunity presented earlier had more severe inflammation and often required surgical intervention [8]. 

Apart from the JIA, we believe that the use of polyglactin suture could have also been participatory in the development and evolution of scleral necrosis despite use of aggressive immunosuppressive therapy. Polyglactin is a synthetic, polyfilament, biodegradable suture known to incite a pronounced granulomatous inflammatory response [9–12]. Its participatory role in SINS has been inconclusively hypothesized, never proven [13]. Considering that the scleral melt was localized to the scleral ports which were sutured and spared the nasal unsutured port, it provides indirect evidence of its potential as an inciting agent. Furthermore, the patient’s tissue tolerated nylon sutures well, which was used for securing the paracentesis and in the subsequent grafting procedure, which also suggests a material-specific hypersensitivity.

SINS is believed to result from immune-mediated vascular occlusion and localized ischemia. Repositioning of healthy conjunctiva over the necrotic area has been shown to aid disease resolution by restoring surface vascular perfusion [14]. In our patient, the relentless progression of scleral melt despite high-dose corticosteroids and pulse cyclophosphamide, coupled with the patient’s young age and the inherent risk of spontaneous globe rupture, necessitated a more definitive approach. A scleral patch graft was undertaken not only to structurally reinforce the thinning sclera but also to achieve disease remission. The addition of a conjunctiva-Tenon flap augmented vascular support, as it has shown to contribute to both inflammation control and long-term tectonic stability of the globe [15]. 

The disease was painless with minimal localized ocular surface inflammation and did not respond to combined pulse therapy of corticosteroids and cyclophosphamide. Removal of the polyglactin suture and application of a scleral patch graft assisted in achieving disease remission.

## Conclusion

Pediatric patients can develop SINS following small-gauge vitrectomy, which can be painless with minimal ocular surface inflammation. Polyglactin sutures may serve as a local immune trigger for SINS and should be avoided when possible, in high-risk individuals. Prompt recognition, aggressive immunosuppressive therapy, and early removal of the polyglactin suture can arrest scleral necrosis. For progressive cases, timely surgical intervention—such as scleral patch grafting—is critical to halting disease progression and preserving long-term ocular integrity.

## Data Availability

No datasets were generated or analysed during the current study.

## Declarations

### Presentation at a meeting

Not done.
